# Diabetes coordinated care: a multidisciplinary approach

**DOI:** 10.1186/1757-1146-4-S1-P17

**Published:** 2011-05-20

**Authors:** Lynne Edmonston Gannon, Leonie Zabarauskas

**Affiliations:** 1 Inner South Community Health Service, St Kilda, Victoria, 3082, Australia

## 

The Inner South Community Health Service diabetes working group was convened in 2001 to improve the management of diabetes and develop a clear model of care for Inner South Community Health Service clients with diabetes. Early initiatives included developing a screening tool, a resource folder, staff training and a short term project to complete a needs analysis and propose a model of care. In 2007 the Inner South Community Health Service diabetes coordinated care model was implemented. This is a comprehensive model that describes the client journey and supporting processes from initial identification through to discharge. Membership of the diabetes working group has included podiatrists, dieticians and nurses. Workers not usually associated with diabetes care have also participated, including: intake worker, family counsellor, mental health worker, physiotherapists, case managers, and occupational therapist. All members received external training to up skill their diabetes knowledge. Mandatory training by this multidisciplinary group was subsequently provided to all staff. The diabetes working group has been the driving force behind the development, evaluation and refinement of the diabetes coordinated care model. The model consists of an initial questionnaire for all new clients completed by the intake worker. If clients express an interest in diabetes, then referral for an assessment is made to the team. The team incorporates any of the working group members and other interested staff, including podiatrists not on the working group. From the assessment clients are offered diabetes information pack, referred to appropriate allied health staff, diabetes educator, diabetes education sessions and diabetes support group. The model has also involved liaison with other external health professionals including general practitioners. Annual follow up is completed by a member of the assessment team. The model has built staff capacity around diabetes, increased resources and services within Inner South Community Health Service and evaluation of the program indicates that there is an increased the capacity of clients to manage their diabetes.

